# Anti-NMDA-R encephalitis: follow-up of 24 months

**DOI:** 10.1590/S1980-57642013DN70300012

**Published:** 2013

**Authors:** Emilia Maria Veloso Soares, Roberta Borges Gomes Kauark, Maria Sheila Guimarães Rocha, Sonia Maria Dozzi Brucki

**Affiliations:** 1 MD, Neurologist, Hospital Santa Marcelina, São Paulo SP, Brazil.; 2 MD, PhD, Neurologist, Hospital Santa Marcelina, São Paulo SP, Brazil.

**Keywords:** Anti-NMDA-R, autoimmune encephalitis, psychiatric symptoms

## Abstract

Anti-N-methyl-D-aspartate receptor (anti-NMDA-R) encephalitis is the second-most-common cause of autoimmune encephalitis, based on epidemiological studies.It has been predominantly described in young females, with prominent psychiatric symptoms, memory loss, decrease in level of consciousness, epilepsy, and central hypoventilation. The condition is commonly associated with mature ovarian teratomas. We describe a video report with a classic presentation of anti-NMDA-R encephalitis in a young patient with no identifiable tumor. Anti-NMDA encephalitis is a recognizable and treatable illness. The prognosis of patients depends on early diagnosis, implementation of appropriate immunomodulatory therapy and, in paraneoplastic cases, complete tumor removal. Clinicians should be wary of this condition, especially when assessing patients with recent onset of psychiatric symptoms unresponsive to antipsychotic treatment.

## Abstract (pt)

A encefalite anti-NMDA é a segunda causa mais comum de encefalite autoimune, com base em estudos epidemiológicos. Foi descrito predominantemente em mulheres jovens, com sintomas psiquiátricos proeminentes, perda de memória , diminuíção do nível de consciência, epilepsia e hipoventilação central. É comumente associada a teratomas ovarianos maduros. Descrevemos uma paciente jovem, com a apresentação clássica da encefalite anti-NMDA, sem tumor identificável. A encefalite anti-NMDA é uma doença tratável e reconhecível. O prognóstico dos pacientes depende do diagnóstico precoce, da implementação da terapia imunomoduladora apropriada e, no caso de paraneoplasia, a remoção completa do tumor. Esta condição deve ser lembrada, especialmente quando Tratam-se de pacientes com início recente de sintomas psiquiátricos que não respondem ao tratamento antipsicótico.

## INTRODUCTION

Anti-N-methyl-D-aspartate receptor (anti-NMDA-R) encephalitis is an immune-mediated syndrome that has been predominantly described in young females, typically presenting with acute behavioral change, psychosis, and catatonia, that evolves to include seizures, memory deficits, dyskinesias, speech problems, and autonomic and breathing dysregulation.^1,2^ It is commonly associated with mature ovarian teratomas. We describe a report with a classic presentation of anti-NMDA-R encephalitis in a young patient with no identifiable tumor, and a follow-up of 24 months.

## CASE REPORT

M.C.L, a 39-year-old, mulatto, São Paulo-born woman, nursing assistant with 8 years' schooling and previously in good health, first showed symptoms of apathy, social withdrawal, irritability and emotional lability in August 2011. After about 1 week, the condition evolved to mutism, although she remained responsive. At this point, she was taken to an emergency room service, where she was administered benzodiazepine initially, and discharged home with a prescription for sertraline 50 mg/day. Approximately one month prior, the subject had suffered emotional stress owing to dismissal from her job.

Her condition progressively worsened and the following day she was admitted to a psychiatric unit. She then evolved with movement disorder and episodes described by her family as 'frights'. Subsequently, she no longer interacted, remaining totally removed from her environment. A transfer to our service and neurological assessment was requested. The medical report from the previous hospital contained a record of treatment with neuroleptics with progressive worsening of the presenting condition, associated with episodes of muscle rigidity. The family reported no occurrence of convulsive fits or fever throughout the period described.

On admission in September 2011, she did not follow instructions or the examiner, and stared blankly into space. She presented with generalized spasticity, orofacial dyskinesias, with blinking movements; besides assuming catatonic postures upon manipulation. Occasionally uttered unintelligible sounds, with some episodes of major tremors affecting the upper limbs, in addition to insomnia and psychomotor agitation. Strength was preserved with active and symmetrical reflexes. Attempts at controlling the agitation and catatonic states using benzodiazepines failed. Evolved with dysautonomia during hospital stay, with alternating periods of hypertension and fever. Causes of subacute encephalopathy were explored and, in the presence of the characteristics outlined above, a suspected diagnosis of Anti-NMDA-R encephalitis was reached.

During admission, the patient underwent lumbar puncture for cerebrospinal fluid (CSF) analysis which revealed pleocytosis with predominant lympho-monocytes (9.6 leukocytes, 90% lymphocytes) while glucose and proteins tested normal. Brain MRI results were also normal. Hematological and biochemical exams, serologies for viral and communicable infections, investigation for collagenoses and auto-antibodies study, were all normal (antinuclear antibodies, anti-DNA, antineutrophil cytoplasmic antibodies, anticardiolipin antibodies, antibodies to herpes simplex), with the exception of anti-thyroglobulin and anti-microsomal antibodies (TPOA >600 and TGA 463) which showed elevated levels, despite exhibiting normal thyroid function. Study for occult neoplasms were performed using imaging exams (CT of the chest, abdomen, and pelvis) and tumor markers, all of which tested negative. Upper gastrointestinal endoscopy with biopsy to test for *Tropheryma whipplei* ruled out Whipple's disease. Electroencephalogram (EEG monitoring during the unresponsive and hyperkinetic phases) showed diffuse disorganization of brain electrical activity.

On 12 September 2011, empirical methylprednisolone pulse therapy was started (1g/d for 5 day) after taking a blood sample for anti-NMBA antibody assaying. The patient evolved with progressive improvement of the condition, cessation of oculomasticatory movements, regained eye contact and attempts to rise from the bed. However, mutism and psychomotor symptoms persisted.

Levels of ant-NMDA antibody tested positive, confirming the diagnosis of anti-NMDA encephalitis. Treatment with human immunoglobulin at 0.4 mg/kg was administered for 5 days along with oral corticoid. The patient evolved with steady improvement of the condition, slowly regaining spoken communication. She was discharged home 2 months after admission, alert, responsive, disoriented in time and space, exhibiting broken speech, absence of orofacial dyskinesias and of tremors in upper limbs, but with a persistent catatonic state. She showed steady improvement over ensuing months, receiving a tapering corticosteroid dose and treatment with monthly cyclophosphamide. The catatonic symptoms gradually resolved, and she showed improved communication, resuming all domestic activities although had irritability and a certain aversion to her children.

Residual deficits included behavioral changes such as impulsiveness, binge eating, hyperorality, disinhibition, confabulations, with repeated discourse and actions. The individual had persistent amnesia for the period from condition onset to recovery. She remains independent for basic activities of daily living but did not return to work. The patient scored 23 points on the Mini-Mental State Examination, dropping most points on attention/calculus and repetition questions as well as on the pentagon drawing task. The patient has shown no signs of relapse over the 24-month follow-up period, and no neoplasms were detected on six-monthly repeat exams.

## DISCUSSION

A syndrome with prominent psychiatric symptoms, memory loss, decreased level of consciousness and central hypoventilation was first described in 2005 in young women with ovarian teratoma and antibodies against an antigen highly expressed in the hippocampus.^3,4^ This antibody was identified in 2007 as the NMDA receptor (NMDAR).^5^ Findings from epidemiological studies suggest that this disorder is the most common cause of autoimmune encephalitis after acute demyelinating encephalomyelitis.^4^

The disorder usually presents with mood and behavioral changes, psychiatric symptoms suggesting schizophrenia or catatonia, decline in level of consciousness, hypoventilation, hyperkinesias, and eventual recovery or death.^4,6^ A stereotypical clinical course occurring in phases is typical. A non-specific flu-like prodrome (subfebrile temperature, headache, fatigue) is always followed by a psychotic stage with bizarre behaviour, disorientation, confusion, paranoid thoughts, visual or auditory hallucinations and memory deficits. Because of these features, a large proportion of patients end up in psychiatric therapy, as occurred with our patient. In the phase that follows, decreased consciousness, hypoventilation, lethargy, seizures, autonomous instability and dyskinesias develop.^7^

Roughly half of this patient group exhibit irregularities on cerebral MRI,^7^ in the form of faint or transient contrast enhancement of the cerebral cortex, overlaying meninges, or basal ganglia. These findings were limited to a single area of the brain, occurring mainly in medial temporal lobes, corpus callosum, and rarely in the brainstem.^9^ Frontotemporal atrophy was noted during the convalescent stage in some patients.^6^ Our patient presented no alterations on MRI. The EEG is pathologically altered in over 90% of individuals with the disease.^7^ Investigation of CSF reveals mild lymphocytic pleocytosis in more than 90% of cases, intrathecal protein increase in 33%, and oligoclonal bands in about 25%.^7^

On average, 60% of patients have tumors, in which the presence of nervous tissue has been proven. The probability of an associated neoplasm is dependent on age and gender. In women, the frequency of predominantly ovarian teratoma was about 62%, while only 22% of men were affected (testicular teratoma and small cell lung cancer).^1,6^ In young females and children, tumors were diagnosed in 31% and 9%, respectively.^8^ Symptoms were found to be more often neurological in children and predominantly psychiatric in adults by an observational cohort study that enrolled 557 patients between 2007 and 2012. However, in the majority of cases the progression of symptoms evolved to a similar syndrome.^4^ The higher frequency of teratomas in Asian patients was a novel finding in the cited study.^4^

The prognosis of patients depends on early diagnosis, implementation of appropriate immunomodulatory therapy and, in paraneoplastic cases, complete tumor removal. Current immunotherapeutic strategies include steroids, plasmapheresis, intravenous immunoglobulins, rituximab, which depletes memory B-cells and cyclophosphamide, which crosses the blood–brain barrier.^7,10^

First-line immunotherapy was often insufficient to control the disease; in the group of patients in the cited study, the addition of second-line immunotherapy was associated with a better outcome compared with those not receiving second-line immunotherapy.^4^ Immunotherapy and tumor removal, if applicable, result in substantial neurological improvement in 81% of patients with anti-NMDAR encephalitis after a median follow-up of 24 months. Moreover, second-line immunotherapy with rituximab, cyclophosphamide, or both, improved the outcome of patients who did not respond to first-line treatment and decreased the occurrence of relapses.^4^ However, 25% of patients suffer from severe neurological deficits or die. Upon recovery, amnesia is evident for the duration of the illness, and there is a risk of relapse of the encephalitic syndrome.^6,7^ The gradual process of clinical recovery may take a long time (sometimes years) but can, in some patients, even be associated with improvement of frontotemporal brain atrophy.^6^ The usefulness of immunotherapy and tumor removal in anti-NMDAR encephalitis has level II-2 evidence.^4^ The rate of mortality was estimated at 7%.^4^

The psychosis and cognitive and behavioral deficits in patients with anti-NMDAR encephalitis most likely result from NMDA receptor hypofunction, directly and indirectly affecting synapse and circuit structure and function in regions that bind NR1 autoantibodies.^10,12^

In conclusion,The anti-NMDA encephalitis case reported was consistent with those reported in the literature, except for the fact that the patient did not present seizures and exhibited serologic presence of thyroid autoantibodies (as an epiphenomenon). Recognition of the combinations of symptoms, which usually include alteration of cognition and behaviour (psychiatric symptoms such as psychosis, catatonia, apathy), abnormal movements (dyskinesias, especially orofacial) and/or new-onset epilepsy, should prompt testing for antibodies against the NMDA receptor. This diagnosis should also be considered in patients with "encephalitis of unknown origin," "drug-induced psychosis", and "encephalitis lethargica".^11^

Anti-NMDA encephalitis is a recognizable and treatable illness, that can be diagnosed serologically. Patients, in general, tend to have good outcomes, particularly with aggressive and prompt therapy. Clinicians should be wary of this condition, especially when assessing patients with recent onset of psychiatric symptoms unresponsive to antipsychotic treatment, bearing in mind that most patients are initially evaluated at psychiatric services. Future studies should clarify the best type and duration of immunotherapy as well as the role of prodromal events in triggering the immune response.
